# The Roles of Mitogen-Activated Protein Kinase Pathways in TGF-**β**-Induced Epithelial-Mesenchymal Transition

**DOI:** 10.1155/2012/289243

**Published:** 2012-01-29

**Authors:** Ting Gui, Yujing Sun, Aiko Shimokado, Yasuteru Muragaki

**Affiliations:** First Department of Pathology, Wakayama Medical University School of Medicine, 811-1 Kimiidera, Wakayama 641-0012, Japan

## Abstract

The mitogen-activated protein kinase (MAPK) pathway allows cells to interpret external signals and respond appropriately, especially during the epithelial-mesenchymal transition (EMT). EMT is an important process during embryonic development, fibrosis, and tumor progression in which epithelial cells acquire mesenchymal, fibroblast-like properties and show reduced intercellular adhesion and increased motility. TGF-**β** signaling is the first pathway to be described as an inducer of EMT, and its relationship with the Smad family is already well characterized. Studies of four members of the MAPK family in different biological systems have shown that the MAPK and TGF-**β** signaling pathways interact with each other and have a synergistic effect on the secretion of additional growth factors and cytokines that in turn promote EMT. In this paper, we present background on the regulation and function of MAPKs and their cascades, highlight the mechanisms of MAPK crosstalk with TGF-**β** signaling, and discuss the roles of MAPKs in EMT.

## 1. Introduction

Signal transduction networks allow cells to perceive changes in the intra- and extracellular environment and respond to them appropriately. Mitogen-activated protein kinase (MAPK) cascades are one of the most thoroughly studied signal transduction systems and have been shown to participate in a diverse array of cellular programs, including cell differentiation, movement, division, and death [1]. MAPKs are serine/threonine kinases that play important roles in a vast array of pathophysiological processes. The family is divided into four main subfamilies: extracellular-regulated kinases (ERKs), Jun N-terminal kinases (JNKs), p38 MAPK, and ERK5. All of these proteins are characterized by the presence of a typical activation module and a conserved activation domain [2]. ERK1 and ERK2 are activated by mitogenic stimuli, whereas JNK and p38 MAPK, which are also called stress-activated protein kinases (SAPKs), are activated by environmental and genotoxic stresses [3–5]. The ERK5 cascade is a MAPK pathway that transmits both mitogenic and stress signals, yet its mechanism of activation is not fully understood [6]. MAPK can be regulated by TGF-*β* stimulation [7], which represents an important mechanism for Smad-independent TGF-*β* signaling. Here, we focus mainly on the cross-talk between MAPK and TGF-*β* signaling.

The TGF-*β* superfamily of signaling molecules controls a diverse set of cellular responses, including cell proliferation, differentiation, extracellular matrix remodeling, and embryonic development. Consequently, when not strictly controlled, TGF-*β* signaling can contribute to the pathogenesis of cancer as well as fibrotic, cardiovascular, and autoimmune diseases [8, 9]. Members of the TGF-*β* superfamily (e.g., TGF-*β*s, activins, and bone morphogenetic proteins (BMPs)) signal via heteromeric serine/threonine kinase transmembrane receptor complexes [10–13]. The effects of TGF-*β* are mediated by three TGF-*β* ligands, TGF-*β*1, 2, and 3 via TGF-*β* type I and II receptors [9, 14, 15]. The binding of the ligand to its primary (type II) receptor, a constitutively active kinase, allows the recruitment, trans-phosphorylation, and activation of the signaling (type I) receptor. The receptor, also known as activin receptor-like kinase 5 (ALK5), is then able to exert its phosphorylation-dependent serine-threonine kinase activity to phosphorylate Smad2 and Smad3 [16–18]. These receptor-activated Smads (R-Smads) interact directly with and are phosphorylated by activated TGF-*β* receptor type I [19, 20]. Smad1, Smad5, and Smad8 are specific substrates of the BMP receptors, whereas Smad2 and Smad3 are activated by both TGF-*β* and activin receptors [17, 21]. Upon phosphorylation, they form heteromeric complexes with Smad4 [22], a common mediator of all Smad pathways. The resulting Smad heterocomplexes are then translocated into the nucleus where they activate target genes by either binding DNA directly or in association with other transcription factors [10, 12, 13, 17, 18]. Members of the third group of Smads, known as inhibitory Smads (Smad6 and Smad7) [23], control Smad signaling by preventing the phosphorylation and/or nuclear translocation of receptor-associated Smads and by inducing receptor complex degradation through the recruitment of ubiquitin ligases [24–26]. More recently, Smad7 was shown to recruit the protein phosphatase complex, type 1 protein serine/threonine phosphatase (PP1), and growth arrest and DNA damage-inducible protein 34 (GADD34) to activated TGF-*β* receptors, stabilizing them and thereby inducing receptor dephosphorylation and deactivation [26]. Following target gene transcription, Smad complexes are released from the chromatin and may undergo ubiquitination and subsequent proteasomal degradation.

These Smad pathways are not the only means by which TGF-*β*s regulate cellular functions. Smad-independent pathways including the mitogen-activated protein kinase (MAPK), nuclear factor *κ*-light chain-enhancer of activated B cells (NF-*κ*B), and PI3 kinase/AKT pathways also participate in TGF-*β* signaling, and these pathways can either be induced by TGF-*β* or modulate the outcome of TGF-*β*-induced Smad signaling [21, 27, 28]. Indeed, broad evidence suggests that Smad signaling is tightly integrated within a complex network of signaling pathways with cross-talk that modify the initial Smad signals and allow the pleiotropic activities of TGF-*β*. There are also instances in which Smad signaling is not required for some TGF-*β* responses, as exemplified by the Smad-independent activation of the cyclin kinase inhibitors p15 and p21 in HaCaT keratinocytes, and the transcriptional activation of the fibronectin promoter via MAPK-dependent mechanisms. It appears clear that Smad proteins are not only the primary substrates for the TGF-*β* receptor kinases but may also be phosphorylated by MAPKs in response to either TGF-*β* itself or to various cytokines. Such R-Smad phosphorylation by MAPKs may serve to regulate Smad by modulating either its transcriptional activity or its capacity to translocate into the cell nucleus [28, 29]. Smad proteins are also capable of physically interacting with transcription factors that are also substrates of MAPKs, adding more complexity to the already intricate relationship between the MAPK and Smad pathways.

The epithelial-mesenchymal transition (EMT) is a complex, stepwise phenomenon that occurs during embryonic development and tumor progression [30]. EMT is also associated with chronic inflammatory and fibrogenic diseases that affect the lungs, the liver, and the peritoneum of patients undergoing peritoneal dialysis [31, 32]. EMT and the reverse process, termed the mesenchymal-epithelial transition (MET), play central roles in embryogenesis, cancer invasion and metastasis, and fibrosis [33, 34]. EMT is characterized by the disruption of intercellular junctions, the replacement of apical-basolateral polarity with front-to-back polarity, and the acquisition of migratory and invasive phenotypes. Cells that have undergone EMT also acquire the capacity to produce extracellular matrix (ECM) components and a wide spectrum of inflammatory, fibrogenic, and angiogenic factors [35]. EMT is triggered by the interplay of several extracellular signals, such as ECM components, soluble growth factors, and cytokines. These signals include members of the TGF-*β* and fibroblast growth factor families, epidermal growth factor, and hepatocyte growth factor [30]. TGF-*β* was first described as an inducer of EMT in normal mammary epithelial cells, and several studies have established crucial roles for TGF-*β*-induced EMT [36].

A key question in studies of MAPK is how a ubiquitously active regulatory enzyme generates a specific and biologically appropriate cellular response during EMT. This paper will summarize some of the latest data from the literature regarding the interactions among MAPK, TGF-*β*, and other factors, with a major focus on the cellular events that contribute to EMT.

## 2. Four Subfamilies of MAP Kinases and Their Substrates in Each Signaling Cascade

**Figure 1:**
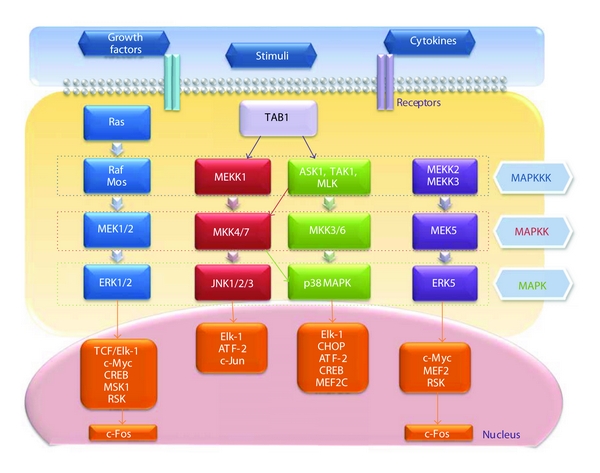
The network of the mitogen-activated protein kinase (MAPK) family. Extracellular stimuli transduce signals to the nucleus. The sequential phosphorylation of MAPKKK, MAPKK, and MAPK activates their nuclear targets, kinases, and transcription factors. For details, refer to the text.

MAP kinases are a large group of proteins that allow numerous extracellular signals to rapidly activate nuclear transcription factors [37] (Figure 1). They consist of at least four subfamilies: the extracellular signal-regulated kinases (ERK1 and ERK2), the stress-activated protein (SAP) kinases, known as c-Jun N-terminal kinases (JNK1, JNK2, and JNK3), the p38 MAPKs (*α*, *β*, *γ*, and *δ*) [2], and ERK5 [38]. ERK5, which is also known as big MAP kinase 1 (BMK1) and has been described as a mediator of Src activation [39], is twice as large as other MAPKs [40].

Signaling initiated by each MAPK pathway occurs through the sequential phosphorylation of a MAPK kinase kinase (MAPKKK), a MAPK kinase (MAPKK), and a MAPK by membrane-associated kinases, such as cytokine or growth factor receptors [41]. MAPK activation leads to the downstream phosphorylation of nuclear kinases or, most commonly, transcription factors. Figure 1 provides a simplified view of the various MAPK pathways and includes most of the MAPK members and substrates cited in the text below.

ERK1 and ERK2, isoforms of the classical MAPK, are phosphorylated by the MAPKKs MEK1 (for MAPK/ERK kinase 1) and MEK2, which are substrates of the MAPKKK Mos and Raf-1 [42]. Raf-1 is activated by the membrane-bound small G-protein Ras following induction by mitogenic stimuli, such as epidermal growth factor (EGF), upon binding and activation of their respective receptors (i.e., EFGR). ERK-mediated pathways are mainly involved in proliferation and differentiation and are generally considered antiapoptotic.

JNK family members are the substrates of MAPK kinase 4 (MKK4, also known as SEK1) and MKK7. p38 MAPK is phosphorylated by MKK3 and MKK6, which are the substrates of apoptosis signal-regulating kinase-1 (ASK1), mixed lineage kinases (MLK), and TGF-*β*-activated kinase-1 (TAK1) [43, 44]. MEK kinase (MEKK1) and TAK1 activate JNK through MKK4 or MKK7 and activate p38 MAPK through MKK3 or MKK6. JNK and p38-signaling pathways are activated by stress stimuli, many of which induce apoptosis, but, in some cellular systems, they have also been implicated in proliferation and differentiation [45, 46].

Upon ERK5 stimulation, two members of the MAPKKK family, MEKK2 and MEKK3, activate MEK5, a MAPKK that is specific for ERK5 [47]. Unlike the first three groups of MAPKs, this pathway has not yet been clearly shown to be activated by TGF-*β* or to interfere with Smad signaling.

MAPK pathways control the cell response to changes in the extracellular environment through the regulation of transcription factors in the nucleus [48]. Thus, to transmit extracellular signals to the nucleus, the terminal components of the MAPK pathways, such as ERK1/2, JNK, and p38 MAPK, must translocate to the nucleus.

A variety of transcription factors and downstream kinases serve as substrates for activated MAPKs [49, 50]. These include activating protein-1 (AP-1), a family of pleiotropic transcription factors comprised of homo- and heterodimers of Fos, Jun, and activating transcription factor (ATF) family members that are involved in the control of cell proliferation, death, and survival, as well as tumorigenesis [51, 52]. Activated ERK1/2 phosphorylates many substrates, including TCF/Elk-1 and c-Myc, and activates cAMP response element binding protein (CREB) and protein kinases, such as mitogen- and stress-activated protein kinase 1 (MSK1) and ribosomal S6 kinase (RSK), which subsequently induces the immediate early gene c-Fos [53, 54].

p38 MAPKs activate many substrates including E twenty six-like transcription factor 1 (Elk-1), CCAAT/enhancer binding protein homologous protein (CHOP), ATF-2, CREB, and myocyte-specific enhancer factor 2C (MEF2C) [55]. JNK is the only MAPK that phosphorylates c-Jun, the main component of AP-1 complexes, and also acts on ATF-2 and Elk-1 [2, 56]. Phosphorylation of c-Jun activates this key member of the AP-1 family of transcription factors, which can then bind the specific AP-1 recognition sites TGAG/CTCA to transactivate target genes [57]. Upon activation, CREB and ATF-2 bind to CRE sites (TGACGTCA) within target gene promoters [58]. Heterodimers of c-Jun and ATF-2 have also been shown to bind to CRE sites [59]. ERK5, similar to ERK1/2, phosphorylates c-Myc, MEF2, and RSK, subsequently inducing c-Fos [60, 61].

## 3. Smad-Dependent and -Independent MAPK Activation by TGF-*β*

TGF-*β* has been shown to activate all ERK, p38 MAPK, and JNK MAPKs in numerous cell types [62–65] through Smad-dependent and -independent transcriptional mechanisms. Because MAPK activation is not a specific feature of TGF-*β* signaling and may be produced by various extracellular stimuli, including cytokines, ultraviolet irradiation, cell-cell or cell-matrix contacts [66–68], the outcome of Smad-dependent or -independent MAPK interactions should be viewed not only as the result of TGF-*β* signaling but also as a consequence of cytokine networks acting in concert to modulate MAPK signals.

**Figure 2:**
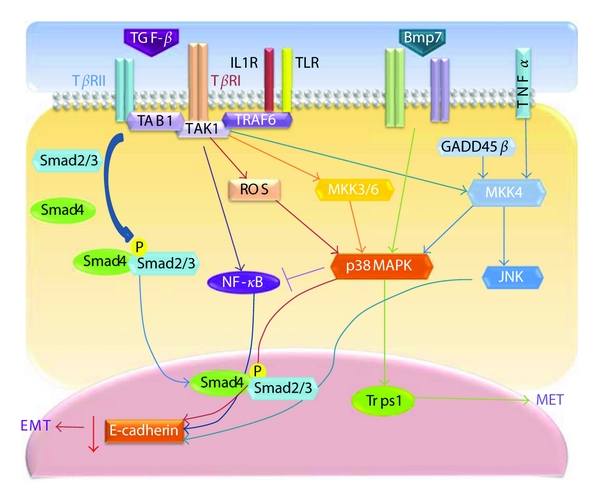
The involvement of JNK and p38 MAPK pathways in the TGF-*β*-induced epithelial-mesenchymal transition. Upon TGF-*β* ligation, the receptor phosphorylates Smad2/3 and interacts with TRAF6, which recruits TAK1 and TAB1 to activate JNK and p38 MAPK. The activated JNK and p38 MAPK can act in a Smad-dependent or -independent manner to regulate EMT by controlling the downstream transcriptional factors. This figure depicts the cross-talk between JNK, p38 MAPK, and TGF-*β* signaling in different cellular systems and illustrates how these networks may function in a stimuli-dependent manner to determine the EMT response.

As an example of Smad-dependent MAPK activation, in mink lung epithelial cells, TGF-*β*-induced activation of JNK mediates Smad3 phosphorylation, which is required for Smad3-dependent transcriptional responses [69] (Figure 2).

However, the initial evidence for Smad-independent activation of MAPK by TGF-*β* came from the observation that the activation of JNK in response to the TGF-*β* pathway was possible in Smad4-deficient cells and cells overexpressing dominant-negative Smads, despite the deficient Smad cascade. It has also been shown that a mutated TGF-*β* type I receptor that cannot phosphorylate R-Smad can still activate p38 MAPK signaling in response to TGF-*β* [70, 71].

**Figure 3:**
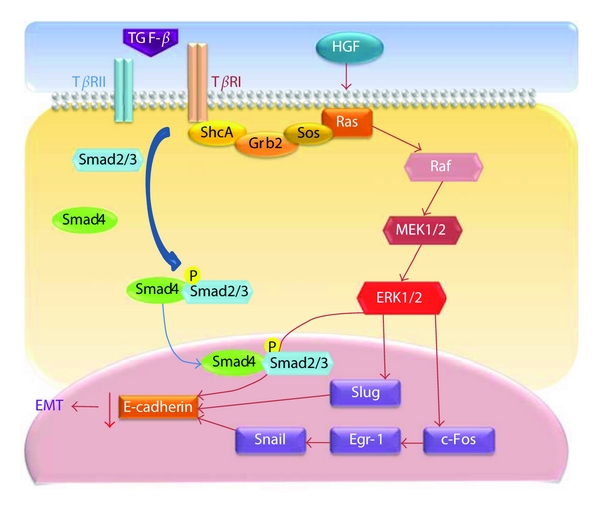
The involvement of the ERK pathway in the TGF-*β*-induced epithelial-mesenchymal transition. After TGF-*β* induces the phosphorylation of serine and/or tyrosine on T*β*RI and/or ShcA, ShcA is capable of recruiting Grb2 and Sos to active ERK1/2 through Ras, Raf, and MEK1/2. The activation of Ras can also be induced by HGF to control the EMT, which is regulated by Snail. This figure depicts the cross-talk between ERK, TGF-*β*, and other factors in different cellular systems and illustrates how these networks may function in a stimuli-dependent manner to determine the EMT response.

Several other Smad-independent signaling examples have been described in the literature. TGF-*β* can activate ERK via rapid activation of Ras in rat intestine [72] (Figure 3). TGF-*β* type I receptor could phosphorylate the ShcA adaptor protein that subsequently associates with Grb2 and Sos in the cytoplasm in the absence of ligand stimulation [73, 74] (Figure 3). The ShcA/Grb/Sos complex is a well-established link between receptor tyrosine kinases and the MEK and ERK pathway via Ras and Raf activation [75].

The mechanisms of ERK, JNK, or p38 MAPK activation by TGF-*β* and the associated biological consequences are not fully characterized. ERK activation by TGF-*β* in epithelial cells may involve Ras signaling [76], while JNK and p38 MAPK signaling could be activated by various MAPKKKs in response to various stimuli. The first MAPKKK known to be activated by TGF-*β* family members was TGF-*β*-activated kinase 1 (TAK1), which was originally identified as a MAPKKK activated by TAB1 (TGF-*β*-activated kinase-binding protein-1) downstream of TGF-*β*/BMP receptors. TAK1 positively regulates the JNK and p38 kinase pathways [77] (Figure 2).

TGF-*β*1 may induce rapid and prolonged activation of p38 MAPK, depending on the cell type. Rapid and transient p38 MAPK activation has been described in certain cell types, including human neutrophils, HEK293, and C2C12 cells, and may be mediated by the induction of TAK1 in an R-Smad-independent manner. On the other hand, the prolonged and sustained p38 MAPK activation observed in pancreatic carcinoma cells, hepatocytes, and osteoblasts requires Smad signaling. Smad activation induces the expression of GADD45*β*, an upstream activator of MKK4, and thus promotes the prolonged activation of p38 MAPK [78] (Figure 2). Functional differences between rapid and prolonged activation of p38 MAPK may be dependent on cell type, but, at least in pancreatic cells, prolonged activation through the Smad-mediated induction of GADD45*β* may contribute to the tumor-suppressive effect of TGF-*β* [78].

## 4. The Association between MAPK and TGF-*β* Signaling in EMT

EMT is a complex process involving a restructuring of the cytoskeleton, cell membrane, and cell-cell junctions. Previous studies have implicated several molecules in different aspects of EMT. However, the aspects of EMT that might be mediated by MAPK signaling have not yet been defined.

ERK activation may be important for several key features of EMT that could cause the loss of epithelial characteristics and acquisition of mesenchymal properties, including the downregulation of adherens junctions and their affiliated proteins (e.g., E-cadherin), increased MMP activity, the induction of actin stress fibers, and the acquisition of motile and invasive properties [79–81]. ERK activation is one of the Smad-independent events that is necessary for TGF-*β*-mediated EMT [82, 83]. ERK is required for the disassembly of cell adherens junctions and the induction of cell motility by TGF-*β*. In a transcriptomic screen of genetic programs for TGF-*β*-induced EMT, TGF-*β*-stimulated ERK activation regulates a subset of target genes, a large proportion of which have defined roles in cell-matrix interactions, cell motility, and endocytosis [82]. These genes are known to function in the remodeling of integrin-based cell-matrix adhesion and in promoting cell motility.

The loss of E-cadherin is a critical step in EMT [84]. There is compelling evidence that ERKs repress E-cadherin expression to drive EMT in many experimental systems [85]. Previous studies have demonstrated that ERK is rapidly activated by TGF-*β* in culture models of EMT, and a specific inhibitor of MEK (upstream of ERK) blocks key morphologic features of EMT, such as the disassembly of E-cadherin-mediated adherens junctions, in various models [86, 87]. Several transcriptional repressors of E-cadherin have now been identified, including two members of the Snail superfamily of the zinc-finger transcription factors, Snail [88] and Slug [89]. Choi et al. found that TGF-*β*1-induced Slug expression was significantly inhibited by MEK- and JNK-specific inhibitors, indicating that MAPK pathways are involved in the regulation of Slug expression by TGF-*β*1 [90].

Recent data suggest that the aberrant activation of ERK may play an important role in diverting the TGF-*β* response towards EMT in kidney epithelial cells. Raf activation confers protection against TGF-*β*-induced apoptosis while enhancing the proinvasive effects of TGF-*β* [91]. Furthermore, the induction of EMT in breast tumor cells is dependent on the presence of both activated Ras and a functional TGF-*β* autocrine loop that is enhanced by Ras [86, 91]. Gene array data obtained from human keratinocytes induced to undergo EMT by TGF-*β* provided the first insights into ERK-dependent gene targets with roles in cell-matrix interactions and cell motility [92].

Perhaps the best-characterized interaction between TGF-*β* and MAPK signaling involves the JNK and p38 MAPK signaling cascades (Figure 2). TGF-*β* can rapidly activate JNK through MKK4 [69, 93] and p38 MAPK through MKK3/6 in various cell lines [70, 94]. Further upstream, MKKs are activated by the MAPKKKs; TAK1 is one of these activating MAPKKKs. Because TAK1 is rapidly induced by TGF-*β*1 and plays a role in p38 MAPK activation and JNK and NF-*κ*B signaling [95], some researchers have focused on TAK1 and found that p38 MAPK maintains E-cadherin expression by suppressing TAK1/NF-*κ*B signaling, thus impeding the induction of EMT in human primary mesothelial cells [96].

TNF receptor associated factor 6 (TRAF6), which plays an important role in the activation of TAK1 in interleukin-1 receptor (IL-1R) and Toll-like-receptor-(TLR-) mediated signaling pathways, was found to be crucial for the TGF-*β*-induced activation of the TAK1-JNK/p38 MAPK pathways [97, 98]. The TRAF6-TAK1-JNK/p38 MAPK pathway plays an important role in TGF-*β*-induced EMT (Figure 2). Inhibiting p38 activity using a p38 inhibitor or dominant-negative forms of MKK3 or p38 impairs TGF-*β*-mediated reorganization of the actin cytoskeleton and results in changes in cell shape [99]. Knocking down TRAF6 expression also inhibits TGF-*β*-mediated EMT [98]. Thus, activation of the TRAF6-TAK1-p38 MAPK pathway is another requirement for TGF-*β*-induced EMT.

Studies of the roles of MAPK family proteins in the genesis of EMT have produced conflicting results, likely due to the heterogeneity of the models and the different experimental approaches used. p38 MAPK appears to promote EMT during development and in tumors [100, 101]. According to an earlier study, p38 MAPK can regulate actin organization via heat shock protein 27 (HSP27) [102]. Therefore, p38 MAPK may function in the TGF-*β*-induced reorganization of the actin cytoskeleton parallel to or upstream of the RhoA/Rock pathway [103]. In addition, p38 MAPK may contribute to the expression of TGF-*β* target genes that are casually involved in EMT because p38 MAPK has been implicated in TGF-*β* transcriptional responses through its activation of ATF2 and Sp1 [104]. Taken together, these results suggest that the MAPK pathway contributes to TGF-*β*-induced changes in the actin cytoskeleton and in cell shape during EMT.

In recent years, significant evidence has indicated that the p38 MAPK pathway is an important intracellular signal transduction pathway in TGF-*β*1-induced EMT in renal tubular epithelial cells [105, 106]. Activated p38 MAPK can directly regulate the protein synthesis of *α*-smooth muscle cell actin (*α*-SMA) and thus indirectly activate the Smad pathway, leading to excessive matrix deposition and finally inducing fibrosis. For example, reactive oxygen species (ROS), which have been shown to mediate TGF-*β*-induced cellular responses in various cells [107], play an important role in EMT in rat proximal tubular epithelial cells, primarily through the activation of MAPK but also indirectly through ERK and subsequently through the phospho-Smad2 pathway [108] (Figure 2).

Semaphorin-4C (Sema4C) is essential for the activation of p38 MAPK [109]. The semaphorins are a large family of secreted or membrane-bound proteins that share a conserved Sema domain, which is known to regulate tumor progression [110], angiogenesis [111], nervous system development [112], and immune cell interactions [113]. Sema4C plays an important role in TGF-*β*1-induced EMT through its activation of p38 MAPK in proximal tubular epithelial cells [114]. Sema4C knockdown strongly inhibits the phosphorylation of p38 and reverses TGF-*β*1-induced EMT. Trps1, an atypical member of the GATA-type family of transcription factors [115], acts downstream of bone morphogenetic protein 7 (BMP7) via p38 [116]. Knockdown of Trps1 or p38 MAPK inhibits the BMP7-induced MET.

 In advanced stages of tumor development, TGF-*β* promotes tumor metastasis by stimulating EMT, matrix metalloproteinase (MMP) expression, and by angiogenesis and inhibiting immune surveillance [117–119]. Numerous studies have revealed that TGF-*β*-induced EMT can be blocked by inhibiting MAPK activation. Synergy between TNF-*α* and TGF-*β* signaling enables p38 MAPK activation to promote the rapid morphological conversion of colon carcinoma epithelia to dispersed cells with mesenchymal phenotypes [85, 120]. Hepatocyte growth factor/scatter factor (HGF) has several functions in the induction of epithelial cell scattering, motility, and tumor progression. One underlying mechanism that could explain this observation is that HGF upregulates Snail, a transcriptional repressor involved in EMT, through MAPK and early growth response factor-1 (Egr-1) (Figure 3).

## 5. Future Perspective and Conclusions

Within the past few years, considerable progress has been made toward understanding the signaling cascades and multiple pathways that involve MAPKs. At present, it is clear that cooperation between TGF-*β*-induced Smad signaling and the MAPK pathway determines the final cellular response to TGF-*β*, especially during EMT. It will not be surprising if more associations between the MAPK pathway and EMT are discovered in the future. The computational and mathematical modeling of biological systems has become increasingly valuable in recent years, and a wide variety of mathematical models of the MAPK pathway have led to some novel insights and predictions about how this system functions [121]. Further cross-talk research will undoubtedly rely on the development of new computational systems and will reveal novel mechanisms that contribute to TGF-*β*-dependent and -independent MAPK signaling, advancing our understanding of how MAPK can induce a plethora of diverse biological responses, including EMT. A major goal will be to determine how the specificity in MAPK downstream signaling is achieved in different cell lines and animal models; this information could be used to seek out clinical advantages in combination therapy.
